# MiR-103a-3p Promotes Zika Virus Replication by Targeting OTU Deubiquitinase 4 to Activate p38 Mitogen-Activated Protein Kinase Signaling Pathway

**DOI:** 10.3389/fmicb.2022.862580

**Published:** 2022-03-04

**Authors:** Haiyan Ye, Lan Kang, Xipeng Yan, Shilin Li, Yike Huang, Rongrong Mu, Xiaoqiong Duan, Limin Chen

**Affiliations:** ^1^Institute of Blood Transfusion, Chinese Academy of Medical Sciences and Peking Union Medical College, Chengdu, China; ^2^The Joint Laboratory on Transfusion-Transmitted Diseases (TTDs) Between Institute of Blood Transfusion, Chinese Academy of Medical Sciences and Nanning Blood Center, Nanning Blood Center, Nanning, China

**Keywords:** MiR-103a-3p, Zika virus, flavivirus, OTUD4, p38 MAPK signaling pathway

## Abstract

**Background:**

MicroRNAs (miRNAs) play critical roles in regulating virus infection and replication. However, the mechanism by which miRNA regulates Zika virus (ZIKV) replication remains elusive. We aim to explore how the differentially expressed miR-103a-3p regulates ZIKV replication and to clarify the underlying molecular mechanism.

**Methods:**

Small RNA sequencing (RNA-Seq) was performed to identify differentially expressed miRNAs in A549 cells with or without ZIKV infection and some of the dysregulated miRNAs were validated by quantitative real time PCR (qRT-PCR). The effect of miR-103a-3p on ZIKV replication was examined by transfecting miR-103a-3p mimic or negative control (NC) into A549 cells with or without p38 mitogen-activated protein kinase (MAPK) inhibitor SB203580 and expression levels of ZIKV NS5 mRNA and NS1 protein were detected by qRT-PCR and Western blot, respectively. The potential target genes for miR-103a-3p were predicted by four algorithms and further validated by mutation analysis through luciferase reporter assay. The predicated target gene OTU deubiquitinase (DUB) 4 (OTUD4) was over-expressed by plasmid transfection or silenced by siRNA transfection into cells prior to ZIKV infection. Activation status of p38 MAPK signaling pathway was revealed by looking at the phosphorylation levels of p38 (p-p38) and HSP27 (p-HSP27) by Western blot.

**Results:**

Thirty-five differentially expressed miRNAs in ZIKV-infected A549 cells were identified by RNA-Seq analysis. Five upregulated and five downregulated miRNAs were further validated by qRT-PCR. One of the validated upregulated miRNAs, miR-103a-3p significantly stimulated ZIKV replication both at mRNA (NS5) and protein (NS1) levels. We found p38 MAPK signaling was activated following ZIKV infection, as demonstrated by the increased expression of the phosphorylation of p38 MAPK and HSP27. Blocking p38 MAPK signaling pathway using SB203580 inhibited ZIKV replication and attenuated the stimulating effect of miR-103a-3p on ZIKV replication. We further identified OTUD4 as a direct target gene of miR-103a-3p. MiR-103a-3p over-expression or OTUD4 silencing activated p38 MAPK signaling and enhanced ZIKV replication. In contrast, OTUD4 over-expression inhibited p38 MAPK activation and decreased ZIKV replication. In addition, OTUD4 over-expression attenuated the stimulating effect of miR-103a-3p on ZIKV replication and activation of p38 MAPK signaling.

**Conclusion:**

Zika virus infection induced the expression of miR-103a-3p, which subsequently activated p38 MAPK signaling pathway by targeting OTUD4 to facilitate ZIKV replication.

## Introduction

As an arthropod-borne single-stranded positive RNA virus belonging to the *Flavivirus* genus in the *Flaviviridae* family (Lanciotti et al., 2008), Zika virus (ZIKV) was first identified from a rhesus monkey in Uganda in 1947 (Dick et al., 1952). There have been at least three outbreaks of ZIKV infection in the past two decades and the most recent ZIKV outbreak occurred in Brazil in 2015. Since then, ZIKV has spread to over 94 countries, which poses a serious global public health threat (Filgueiras et al., 2021). Although approximately 80% of individuals infected with ZIKV are asymptomatic (Patterson et al., 2016), it is noteworthy that accumulating evidence suggests that ZIKV infection is associated with neurologic disorders, such as microcephaly in infants and Guillain-Barré syndrome (GBS) in adults (Filgueiras et al., 2021). Up till now, no vaccine or specific anti-viral drugs are licensed for prevention or treatment of ZIKV infection (Mwaliko et al., 2021). One of the main reasons lies in the fact that the pathogenesis of ZIKV infection and how this virus evades host immune response have not been fully elucidated.

P38 mitogen-activated protein kinases (MAPKs) are a class of serine/threonine protein kinases that play critical roles in various cellular process, including virus infection (Chander et al., 2021). Upon stimulation, p38 MAPK is phosphorylated by upstream kinases mitogen-activated protein kinase kinase 3 (MKK3) or mitogen-activated protein kinase kinase 6 (MKK6) and subsequently causes cascade phosphorylation of substrates, including MAPK activated protein kinase 2 (MAPKAPK2) and heat shock protein 27 (HSP27; Kostenko and Moens, 2009; Cuadrado and Nebreda, 2010). It has been reported that p38 MAPK can be activated by several viruses, including hepatitis C virus (HCV), herpes simplex virus type 1 (HSV-1), severe fever with thrombocytopenia syndrome virus (SFTSV; Cheng et al., 2020) enterovirus 71 (EV71; Peng et al., 2014), severe acute respiratory syndrome coronavirus 2 (SARS-CoV-2; Grimes and Grimes, 2020; Ma et al., 2020), and ZIKV (Zhu et al., 2017; Muthuraj et al., 2021). Interestingly, data from previous studies indicated that some viruses, such as hepatitis B virus (HBV), HSV-1, respiratory syncytial virus (RSV), and influenza A virus (IAV), can utilize the p38 MAPK to benefit their replication (Chander et al., 2021), which makes p38 MAPK inhibitor as a promising therapeutic approach for virus infection.

OTU deubiquitinase 4 (OTUD4) is a member of OTD deubiquitinase (DUB) family, which participates in various physiological and pathological processes, such as embryonic development of zebrafish, repair of DNA alkylation damage, and innate immune response (Tse et al., 2009; Zhao et al., 2015, 2018; Liuyu et al., 2019). Zhao et al. (2018) revealed that OTUD4 negatively modulated TLR-mediated NF-κB activation and silencing of OTUD4 increased the phosphorylation of p38 MAPK upon the stimulation of IL-1β. Nevertheless, the role of OTUD4 and p38 MAPK signal pathway in ZIKV replication has not been elucidated.

MicroRNAs (miRNAs) are a class of small endogenous non-coding RNAs with a length of approximately 22 nucleotides. MiRNAs can post-transcriptionally inhibit the expression of their target genes through degrading mRNAs or inhibiting their translation following the base complementary pairing between miRNAs seed sequence and specific mRNAs sequence of target genes, primarily in the 3′-untranslated regions (3′UTRs; Bartel, 2004). Many miRNAs have been reported to engage in ZIKV infection (Estevez-Herrera et al., 2021). For example, miR-142-5p was found to be downregulated by ZIKV infection, while miR-142-5p overexpression suppressed ZIKV replication (Seong et al., 2020). Zhang et al. (2019a) demonstrated that ZIKV infection increased miR-9 expression and decreased its target gene GDNF, which were associated with microcephaly in mice. Shukla et al. (2021) demonstrated that miR-146a induced by ZIKV-NS1 protein suppressed cellular antiviral response in human microglial cells. MiRNAs have also been involved in the regulation of p38 MAPK signaling pathway. MiR-744, miR-124a, and miR-24 could effectively inhibit the phosphorylation of p38 MAPK in influenza A virus infected cells (McCaskill et al., 2017). In this study, we found that ZIKV infection induced miR-103a-3p expression which stimulates ZIKV replication and activation of p38 MAPK signaling pathway, while blocking p38 MAPK signaling suppressed ZIKV replication. Mechanistically, we identified OTUD4 as a direct target gene for miR-103a-3p. Increased miR-103a-3p expression leads to downregulation OTUD4 to stimulate ZIKV replication through the activation of p38 MAPK signaling pathway. Therefore, miR-103a-3p may be one of the host genes exploited by ZIKV to benefit its replication in host cells. Targeting miR-103a-3p or p38 MAPK signaling may be a viable approach for the better control of ZIKV infection.

## Materials and Methods

### Cell Culture and Zika Virus

A549 and 293T cells were obtained from West China Hospital, Sichuan University. The cells were grown in Dulbecco’s modified Eagle’s medium (DMEM; Hyclone, United States) supplemented with 10% fetal bovine serum (FBS; Biological Industries, Israel) and 1% mycoplasma prevention reagent (Transgen, China) at 37°C with 5% CO_2_ in a humidified incubator. ZIKV (GZ01 strain) was generously provided by Dr. Chengfeng Qin (Beijing Institute of Microbiology and Epidemiology, China). ZIKV was propagated and tittered in A549 cells as previously described (Wang et al., 2020). ZIKV stock was stored in aliquots at −80°C freezer for further use.

### Small RNA Sequencing and Data Analysis

A549 cells with or without ZIKV infection by multiplicity of infection (MOI) of 0.5 were harvested at 48 h post-infection for analysis of intracellular miRNAs. The preparation of libraries and the procedure of sequencing were performed by Novogene Co., Ltd (Beijing, China). Briefly, 3 μg total RNA per sample was processed for the small RNA library. Sequencing libraries were generated using NEBNext® Multiplex Small RNA Library Prep Set for Illumina® (NEB, United States) followed by products purification on 8% polyacrylamide gel to isolate the 140–160 bp DNA fragments. Library quality was assessed on a DNA High Sensitivity Chip using the Agilent Bioanalyzer 2100 system (Agilent Technologies, United States). Next, small RNA sequencing (RNA-Seq) was performed on an Illumina Hiseq 4,000 platform and 50 bp single-end reads were generated.

The small RNA-Seq data analysis was performed by Novogene Co., Ltd (Beijing, China). Raw data was assessed by internal quality control and filtered by several criteria to obtain clean reads. Reads were then aligned to the human reference genome using Bowtie (Langmead et al., 2009). The DESeq R package (1.8.3) was used to identify differentially expressed miRNAs following ZIKV infection. The *p*-values were adjusted using the Benjamini & Hochberg method to control the false-discovery rate. An adjusted *p* < 0.05 was deemed differentially expressed miRNAs. The complete raw small RNA sequencing data have been deposited in NCBI’s Gene Expression Omnibus (GEO) with the accession number of GSE146423.^1^

### ZIKV Infection and Treatment of Cells With P38 MAPK Inhibitor

A549 cells were infected with ZIKV by MOI of 0.5 for 4 h at 37°C. After 4 h virus incubation, the medium was aspirated. Cells were washed three times with phosphate-buffered saline (PBS; Hyclone, United States) and then replenished with fresh medium. p38 MAPK inhibitor SB203580 (APExBIO, United States) was dissolved in dimethyl sulfoxide (DMSO) to prepare a 10 mM stock solution and stored at −20°C freezer for further use. SB203580 stock was diluted to indicated concentration in cell culture medium and the cells were pretreated 1 h before ZIKV infection.

### Transfection of Cells With MiR-103a-3p Mimic, siRNA, Plasmid DNA

MiR-103a-3p mimic and negative control (NC) were purchased from RiBoBio (RiBoBio Inc., China) and transfected into A549 or 293T cells at a final concentration of 10 nM using RNAiMAX reagent (Invitrogen, United States) according to the manufacturer’s instructions. Small interfering RNA targeted OTUD4 (GGAACUAGACACGUUGGAATT) and a NC siRNA (UUCUCCGAACGUGUCACGUTT) were chemically synthesized by Sangon Biotech (Sangon Biotech, China). Transfection of siRNA at a final concentration of 20 nM was performed using the RNAiMAX regent according to the manufacturer’s recommended procedures. Recombinant plasmid expressing OTUD4 was constructed by cloning the amplified human full-length OTUD4 cDNA into the expression vector P3 × FLAG-CMV-7.1 (Fenghbio, China). The correct insertion of the OTUD4 gene was verified by DNA-sequencing. The recombinant OTUD4 plasmid and the corresponding empty vector were transiently transfected into A549 cells at a final concentration of 1 μg/ml using the Lipofectamine 3000 reagent (Invitrogen, United States) following the manufacturer’s instructions.

### RNA Isolation, Reverse Transcription, and qRT-PCR

Total cellular RNA was extracted using Trizol reagent (Invitrogen, United States). The first-strand cDNA was synthesized from 1 μg of total RNA by reverse transcription using ReverTra Ace®qPCR RT Master Mix (Toyobo, Japan) according to the manufacturer’s protocol. To validate the differentially expressed miRNAs induced by ZIKV infection, we use miRNAs specific primers (Table 1). The qRT-PCR analysis was carried out using SYBR Green Real-time Master Mix (Novoprotein, China) on a CFX96 Real-Time System (Bio-Rad, United States). The mRNA level of each gene was calculated by using 2^-△△Ct^ method, normalized to GAPDH or U6. The sequences of primers used in this study are listed in Table 1.

**Table 1:** qRT-PCR primer sequences.

Gene name		Primer sequences (5’-3’)
miR-103a-3p	RT	GTCGTATCCAGTGCAGGGTCCGAG GTATTCGCACTGGATACGACTCATAG
	Forward	TTAGCAGCATTGTACAGGG
miR-4454	RT	GTCGTATCCAGTGCAGGGTCCGAG GTATTCGCACTGGATACGACTGGTGC
	Forward	TTAGGATCCGAGTCACGG
miR-203a-3p	RT	GTCGTATCCAGTGCAGGGTCCGAG GTATTCGCACTGGATACGACCTAGTG
	Forward	GTGAAATGTTTAGGACCACT
miR-203b-5p	RT	GTCGTATCCAGTGCAGGGTCCGAG GTATTCGCACTGGATACGACTGTGAA
	Forward	CAGGTAGTGGTCCTAAACATT
miR-210-3p	RT	GTCGTATCCAGTGCAGGGTCCGAG GTATTCGCACTGGATACGACTCAGCC
	Forward	TGTGCGTGTGACAGCG
miR-495-3p	RT	GTCGTATCCAGTGCAGGGTCCGAG GTATTCGCACTGGATACGACAAGAAG
	Forward	CGAAACAAACATGGTGCA
miR-543	RT	GTCGTATCCAGTGCAGGGTCCGAG GTATTCGCACTGGATACGACAAGAAG
	Forward	GAAACATTCGCGGTGC
miR-654-5p	RT	GTCGTATCCAGTGCAGGGTCCGAG GTATTCGCACTGGATACGACGCACAT
	Forward	TGGTGGGCCGCAGAA
miR-493-5p	RT	GTCGTATCCAGTGCAGGGTCCGAG GTATTCGCACTGGATACGACAATGAA
	Forward	TGGTTGTACATGGTAGGCTT
miR-376c-3p	RT	GTCGTATCCAGTGCAGGGTCCGAG GTATTCGCACTGGATACGACACGTGG
	Forward	TCGGAACATAGAGGAAATTC
miRNA	Reverse	GCAGGGTCCGAGGTATTC
OTUD4	Forward Reverse	CTAACTCCTGCGGTGCCTTCTT GCTGAATCAGGTCCAGTGGTCA
GZ01-NS5	Forward Reverse	CCTTGGATTCTTGAACGAGGA AGAGCTTCATTCTCCAGATCAA
U6	Forward Reverse	CTCGCTTCGGCAGCACA AACGCTTCACGAATTTGCGT
GAPDH	Forward Reverse	GCCTCCTGCACCACCAACTG ACGCCTGCTTCACCACCTTC

### Western Blotting and Antibodies

Total protein of cells was extracted by radioimmune precipitation assay (RIPA) lysis buffer containing PMSF protease inhibitor (Biosharp, China) and phosphatase inhibitor cocktail (Transgen, China). The lysates were centrifugated at 15,000 *g* for 20 min at 4°C and the supernatant was collected. Protein concentrations were quantified by BCA Protein Assay Kit (Beyotime, China). A total of 30 μg protein was boiled at 98°C for 5 min and loaded into SDS-PAGE gels. Then the separated protein bands were transferred to a PVDF membrane (Millipore, United States). The membrane was blocked with 5% bovine serum albumin (BSA) (Solarbio, China) at room temperature for 2 h, and then incubated with specific primary antibody at 4°C overnight. The primary antibodies used were as follows: anti-GADPH (Proteintech, China), anti-β-actin (Proteintech, China), anti-FLAG (Sigma, United States), anti-p-p38 MAPK phosphorylated Thr180/Tyr182 (Cell Signaling Technology, United States), anti-p38 MAPK (Cell Signaling Technology, United States), anti-p-HSP27 phosphorylated Ser82 (Cell Signaling Technology, United States), anti-HSP27 (Proteintech, China), anti-ZIKV NS1 (GeneTex, United States), and anti-OTUD4 (Proteintech, China). After washing with Tris-buffered saline with 0.1% Tween-20 (TBST), the membrane was incubated with horseradish peroxidase (HRP)-conjugated goat anti-mouse IgG or anti-rabbit IgG secondary antibodies (Proteintech, China) at room temperature for 1 h. Finally, the membrane was washed and incubated with chemiluminescent HRP substrate (Milipore, United States) to detect the immunoreactive bands using a ChemiDoc imaging system (Bio-Rad, United States). Densitometry was performed with ImageJ software.

### Bioinformatic Analysis of miR-103a-3p Target Genes

The putative miR-103a-3p targets were predicted by four different algorithms, including PicTar,^2^ TargetScan,^3^ miRanda,^4^ and miRMap.^5^ Venn’s diagram was drawn by Venny2.1.^6^

### Luciferase Reporter Assay

The putative binding sites between OTUD4 3′UTR and miR-103a-3p seed sequence were predicted by Targetscan. Wild-type or mutant OTUD4 3′UTR sequences that containing the binding sites of miR-103a-3p seed sequence were constructed into the 3′UTR of the Renilla luciferase gene in the dual-luciferase reporter vector pmiR-RB-Report™ (Ribobio, China). The reporter plasmid also included a firefly luciferase gene, which was used as a reference for normalization. The reporter plasmids were co-transfected with miR-103a-3p mimic or negative control into 293T cells using Lipofectamine 3000 transfection reagent (Invitrogen, United States). At 48 h post-transfection, the cells were lysed for Firefly and Renilla luciferase measurement by dual-luciferase reporter assay (Promega, United States) according to the manufacture’s protocol. The luciferase activities were measured on EnSpire Multimode Plate Reader (PerkinElmer, United States). The Renilla luciferase/Firefly luciferase activity ratio was calculated to determine the binding between miR-103a-3p and cloned 3′UTR of OTUD4.

### Cell Viability Assay

A549 cells were seeded in 96-well plates and treated with SB203580 or transfected with miR-103a-3p mimic, OTUD4 plasmid, OTUD4 siRNA and corresponding negative controls. After 48 h post treatment or transfection, cell viability was detected by Cell Counting Kit 8 (CCK8; Biosharp, China) according to the manufacture’s instruction. In brief, 10 μl CCK8 solution was added to each well and incubated for 2 h at 37°C. Sample absorbance was determined at 450 nm on EnSpire Multimode Plate Reader (PerkinElmer, United States). The cell viability was calculated by using (OD_experiment_ – OD_blank_)/(OD_control_ – OD_blank_) × 100%.

### Statistical Analysis

Statistical analyses and calculations were performed with GraphPad Prism 8 software. All the data presented are representative of at least three independent experiments, unless stated otherwise. Data are expressed as mean ± SD. A two-tailed Student’s *t*-tests were performed to determine the difference, and value of *p* < 0.05 was considered statistically significant.

## Results

### Differentially Expressed miRNAs in ZIKV-Infected A549 Cells

**Figure 1:**
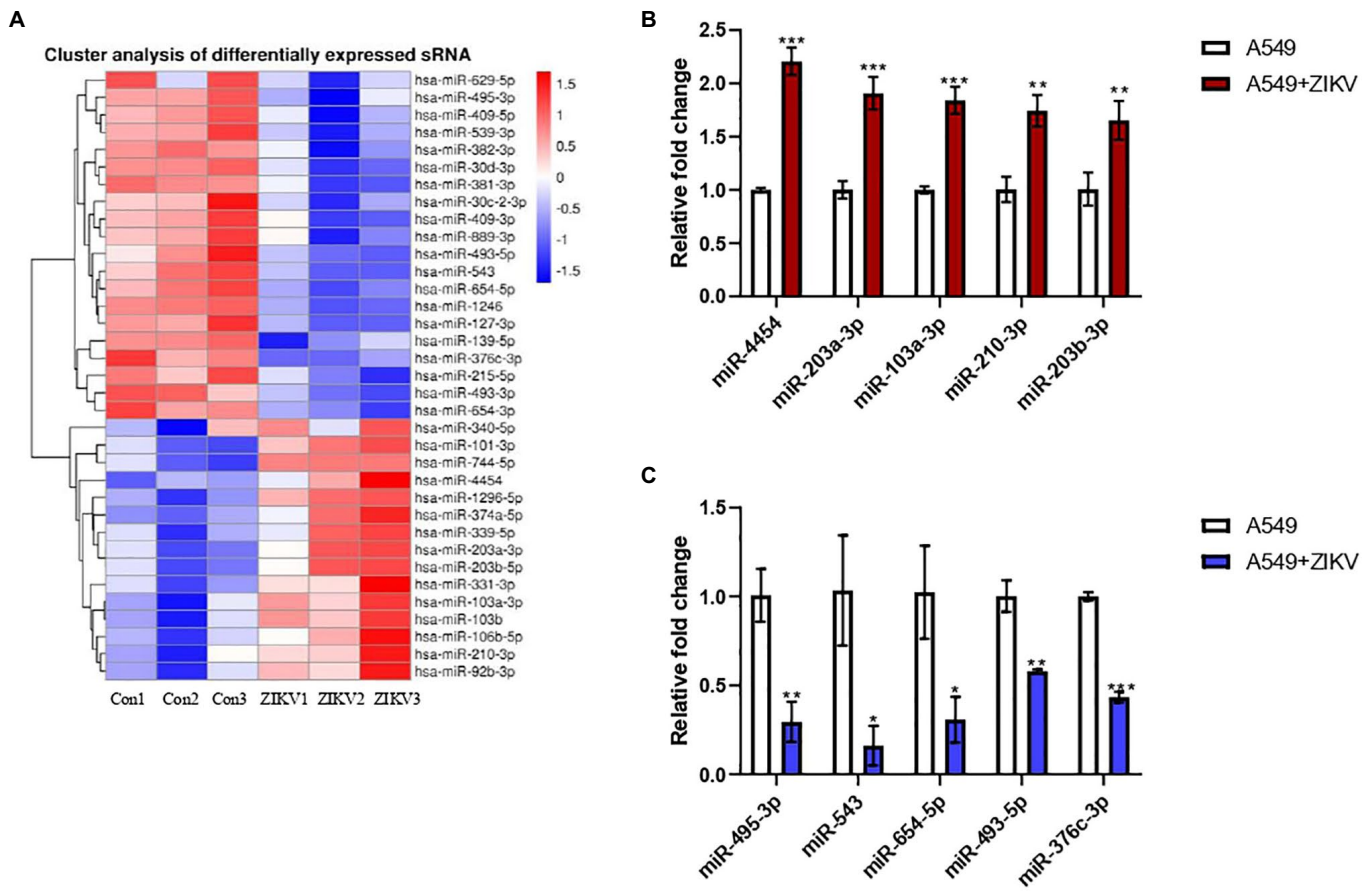
Differentially expressed host MicroRNAs (miRNAs) between A549 and Zika virus (ZIKV) infected A549 cells. A549 cells were infected with ZIKV infection by multiplicity of infection (MOI) of 0.5 and the infected cells were harvested at 48 h post-infection for miRNA profiling by RNA sequencing (RNA-seq) and the data were compared with those obtained from un-infected A549 cells. **(A)** Heat map analysis of the miRNAs expression. Red indicates upregulation and blue indicates downregulation. **(B)** Five upregulated candidate miRNAs were selected to verify by qRT-PCR. **(C)** Five downregulated candidate miRNAs were selected to verify by qRT-PCR. Data are normalized to U6 and presented as mean ± SD. Error bars indicate SD (*n* ≥ 3). ^*^p < 0.05, ^**^p < 0.01, and ^***^p < 0.001.

Our previous study demonstrated that ZIKV could efficiently infect A549 cells (Liao et al., 2020). To investigate the miRNA expression profiles following ZIKV infection, we performed small RNA-seq in A549 cells with or without ZIKV infection. Around 35 dysregulated miRNAs were identified in A549 cells following ZIKV infection compared to uninfected cells (*p* < 0.05; Figure 1A). Five upregulated and five downregulated miRNAs were selected for further validation by qRT-PCR. As shown in Figures 1B,C, the qRT-PCR results for these miRNAs were consistent with the RNA-seq results. Among these dysregulated miRNAs, miR-103a-3p was highly induced by ZIKV infection and our result is supported by most recent study that miR-103a-3p was upregulated in extracellular vesicles (EVs) during ZIKV infection (Tabari et al., 2020). Up till now, no studies on the effect of miR-103a-3p on viral replication have been reported; therefore, we choose it for further investigation using ZIKV as a model virus.

### MiR-103a-3p Stimulates ZIKV Replication

**Figure 2:**
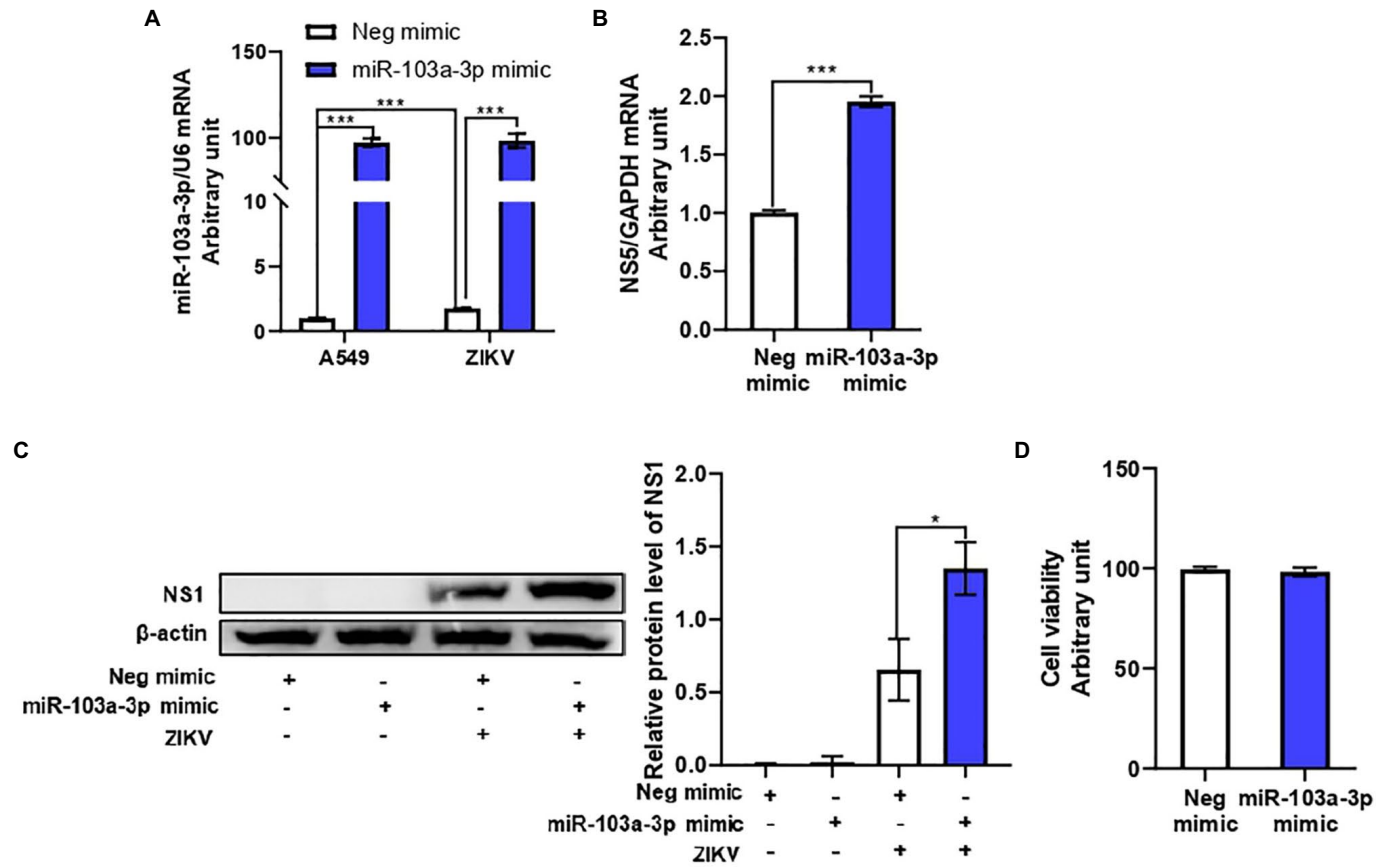
miR-103a-3p stimulates ZIKV replication in A549 cells. A549 cells were transfected with miR-103a-3p mimic or corresponding negative control at a final concentration of 10 nM and infected with ZIKV at a MOI of 0.5. Total RNA and protein of cells were harvested at 48 h post infection for qRT-PCR and Western blot analysis, respectively. **(A)** Level of miR-103a-3p over-expression. **(B)** MiR-103a-3p over-expression promotes ZIKV NS5 RNA expression. **(C)** MiR-103a-3p over-expression enhances ZIKV NS1 protein level. **(D)** miR-103a-3p mimic or negative control transfection does not affect cell viability. Data are presented as mean ± SD. Error bars indicate SD (*n* ≥ 3). ^*^p < 0.05 and ^***^p < 0.001.

In order to determine the effect of miR-103a-3p on ZIKV replication, we transfected miR-103a-3p mimic or the corresponding negative control into A549 cell. As shown in Figure 2A, miR-103a-3p was successfully over-expressed in A549 cells with or without ZIKV infection following transfection. MiR-103a-3p over-expression significantly promoted ZIKV RNA replication and ZIKV NS1 protein expression (Figures 2B,C). In addition, we confirmed that either miR-103a-3p mimic or negative control transfection had no effect on cell viability (Figure 2D). Taken together, these results indicate that ZIKV infection induced miR-103a-3p expression to promote its replication both at mRNA and protein levels.

### MiR-103a-3p Stimulates ZIKV Replication Through Activation of the p38 MAPK Signaling Pathway

**Figure 3:**
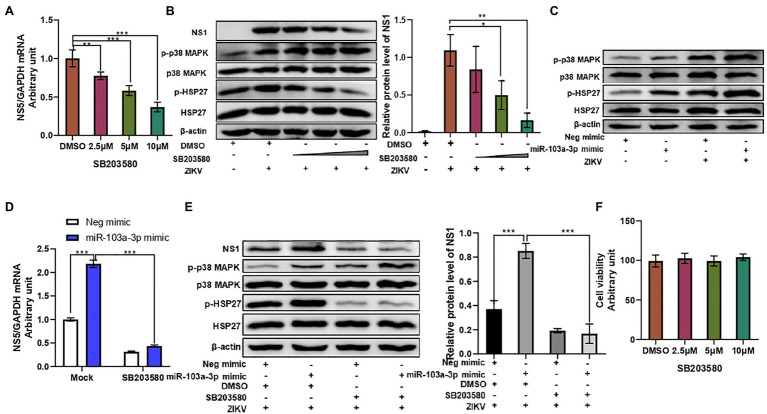
miR-103a-3p stimulates ZIKV replication through activation of p38 MAPK signaling pathway. A549 cells were pretreated with SB203580 at indicated dose for 1 h or transfected with miR-103a-3p mimic or corresponding negative control and infected with ZIKV (MOI = 0.5) for 48 h. Total RNA and protein of cells were harvested at 48 h post infection for qRT-PCR or Western blot analysis. **(A)** SB203580 inhibits ZIKV RNA replication in a dose-dependent manner. **(B)** SB203580 inhibits ZIKV NS1 expression and p-HSP17 but increases p-p38 MAPK protein levels in a dose-dependent manner. **(C)** Over-expression of miR-103a-3p increases p-p38 MAPK and p-HSP27 protein levels. **(D)** SB203580 treatment attenuates the stimulating effect of miR-103a-3p on ZIKV RNA replication. **(E)** SB203580 abrogates the stimulating effect of miR-103a-3p on ZIKV NS1 and p-HSP27, but not of p-p38 MAPK protein levels. **(F)** The selected dose of SB203580 does not affect cell viability. Data are presented as mean ± SD. Error bars indicate SD (*n* ≥ 3). ^*^p < 0.05, ^**^p < 0.01, and ^***^p < 0.001.

Many viruses have been reported to utilize p38 MAPK in order to facilitate their replication (Chander et al., 2021). We next move forward to investigate whether p38 MAPK signaling pathway is involved in miR-103a-3p regulation of ZIKV infection. Firstly, we confirmed that p38 MAPK signaling pathway was activated upon ZIKV infection in A549 cells. As shown in Supplementary Figures S1A,B, increased phosphorylation of p38 MAPK was observed in ZIKV-infected A549 cells both in time-dependent and MOI-dependent manners. Blocking p38 MAPK pathway using SB203580 inhibited ZIKV RNA replication and ZIKV NS1 protein expression in a dose-dependent manner (Figures 3A,B). SB203580 treatment alone did not affect miR-103a-3p expression in A549 cells, but slightly inhibited the expression of miR-103a-3p induced by ZIKV infection (Supplementary Figure S2). Consistent with the previous study (Kumar et al., 1999), SB203580 inhibited the phosphorylation of HSP27 (down-stream of p38 MAPK pathway) but increased p38 MAPK phosphorylation slightly (Figure 3B). Next, we found miR-103a-3p over-expression activated p38 MAPK signaling pathway. The phosphorylation levels of p38 MAPK and HSP27 were significantly increased following miR-103a-3p upregulation in A549 cells with or without ZIKV infection (Figure 3C). SB203580 treatment attenuated the stimulating effect of miR-103a-3p on ZIKV RNA replication and ZIKV NS1 expression, as well as the induction of p-HSP27 (Figures 3D,E). Besides, we did not observe significant effect of SB203580 treatment on cell viability (Figure 3F). Collectively, these results suggest that miR-103a-3p stimulates ZIKV replication through activation p38 MAPK signaling pathway.

### OTUD4 Is a Target of miR-103a-3p

**Figure 4:**
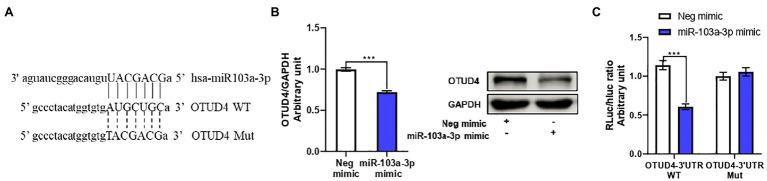
OTU deubiquitinase 4 (OTUD4) is a target gene of miR-103a-3p. A549 cells were transfected with miR-103a-3p or co-transfected with miR-103a-3p and luciferase reporter plasmid. Total RNA, protein, and lysates of cells were harvested at 48 h post transfection for qRT-PCR, Western blot, and Luciferase reporter assays. **(A)** The putative binding sites in wide-type (WT) and corresponding mutant (MUT) sites of miR-103a-3p seed sequence on the 3′-untranslated region (3′UTR) of OTUD4. **(B)** miR-103a-3p decreases both mRNA and protein levels of OTUD4. **(C)** The luciferase activity is decreased in the group co-transfected of miR-103a-3p mimic and OTUD4-3′UTR-WT plasmid, but not of OTUD4-3′UTR-Mut plasmid. Data are presented as mean ± SD. Error bars indicate SD (*n* ≥ 3). ^***^p < 0.001.

After, we clarified miR-103a-3p stimulated ZIKV replication through activation of the p38 MAPK signaling pathway, we next moved on to investigate the target gene of miR-103a-3p. We employed four different prediction algorithms to predict the possible target genes of miR-103a-3p and 177 genes overlapped in all the four algorithms were obtained (Supplementary Figure S3). Among these genes, OTUD4 was closely relevant to host antiviral response and p38 MAPK signaling pathway according to previously studies (Zhao et al., 2018; Liuyu et al., 2019). Interestingly, one possible binding site for miR-103a-3p at the 3′UTR region of OTUD4 was identified (Figure 4A). By qRT-PCR and Western blot, we found OTUD4 expression was significantly downregulated by over-expression of miR-103a-3p compared to negative control (Figure 4B). To further confirm whether OTUD4 is a direct target gene of miR-103a-3p, we performed mutation analysis by dual-luciferase assays. As expected, miR-103a-3p significantly repressed Renilla/Firefly luciferase activity when co-transfected with the wild-type OTUD4 3′UTR plasmid but not of the 3′UTR mutant plasmid (Figure 4C). Taken together, these results support that OTUD4 is a target gene of miR-103a-3p.

### OTUD4 Inhibits ZIKV Replication Through Suppression of p38 MAPK Signaling Pathway

**Figure 5:**
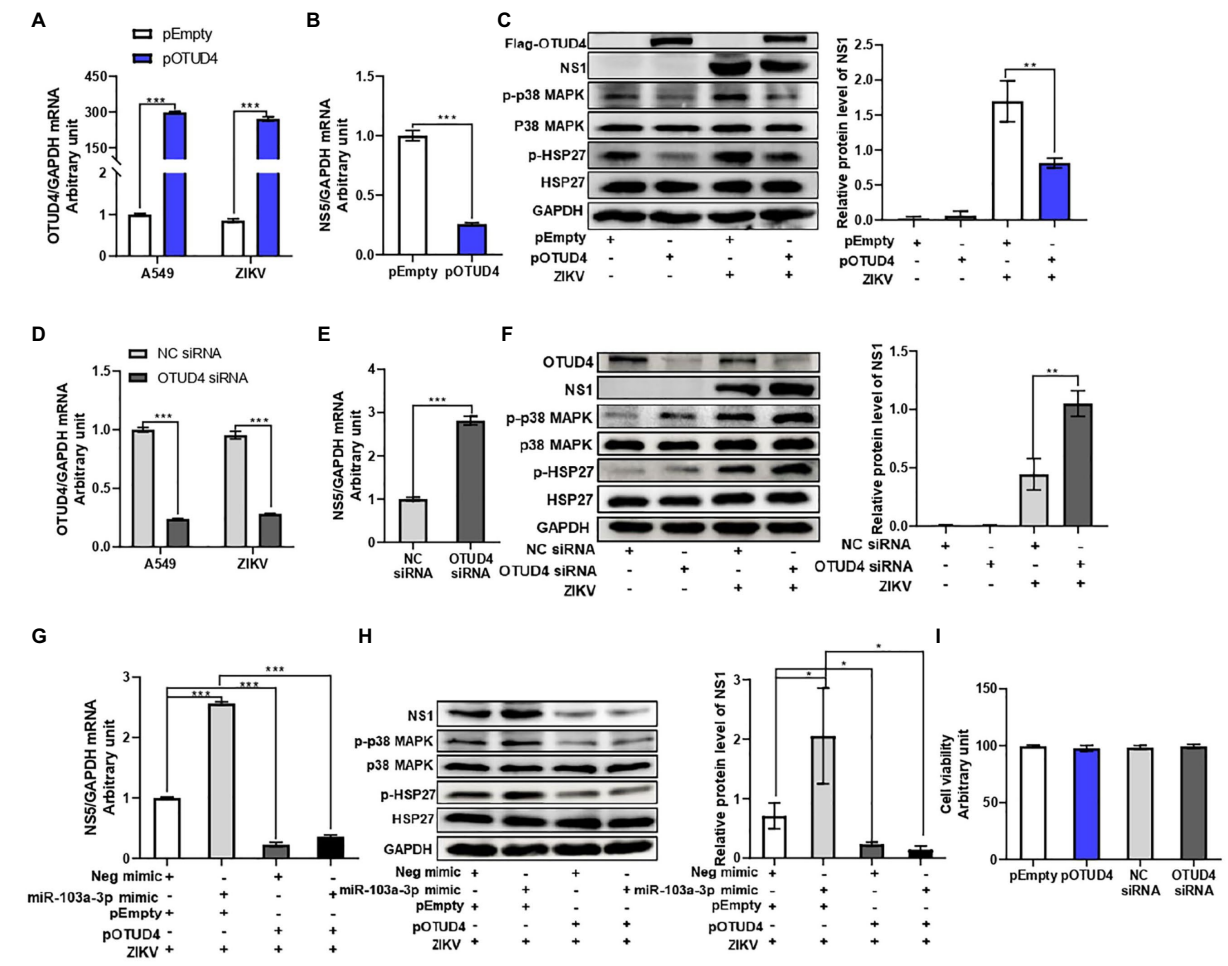
OTU deubiquitinase 4 regulates ZIKV replication through activating p38 MAPK signaling pathway. pOTUD4, OTUD4 siRNA and corresponding control were transfected into A549 cells and then cells were infected ZIKV (MOI = 0.5). Total RNA and protein of cells were harvested at 48 h post infection for qRT-PCR and Western blot analysis, respectively. **(A)** The mRNA expression level of OTUD4 is significantly increased following pOTUD4 transfection compared to pEmpty. **(B)** OTUD4 over-expression inhibits ZIKV NS5 mRNA expression compared to pEmpty. **(C)** Over-expression of OTUD4 decreases ZIKV NS1, p-p38 MAPK, and p-HSP27 protein levels compared to pEmpty. **(D)** The mRNA expression level of OTUD4 is inhibited by OTUD4 siRNA transfection compared to NC siRNA. **(E)** OTUD4 knock-down significantly promotes ZIKV RNA expression compared to NC siRNA. **(F)** Silencing of OTUD4 increases ZIKV NS1, p-p38 MAPK, and p-HSP27 protein levels compared to NC siRNA. **(G)** Over-expression of OTUD4 abrogates the stimulating effect of miR-103a-3p on ZIKV RNA replication. **(H)** OTUD4 over-expression reduces the promoting effect of miR-103a-3p on ZIKV NS1, p-p38 MAPK, and p-HSP27 protein levels. **(I)** pOTUD4 and OTUD4 siRNA transfection into A549 cells did not affect cell viability. Data are presented as mean ± SD. Error bars indicate SD (*n* ≥ 3). ^*^p < 0.05, ^**^p < 0.01 and ^***^p < 0.001.

Next, we sought to explore the regulatory effect of OTUD4 on ZIKV replication. OTUD4 was over-expressed by transfection with Flag-OTUD4 plasmid. Transfection of OTUD4 plasmid led to a remarkable upregulation of OTUD4 mRNA and protein expression in A549 cells with or without ZIKV infection (Figures 5A,C). OTUD4 over-expression significantly inhibited ZIKV RNA replication and ZIKV NS1 protein level compared with empty vector group (Figures 5B,C). Additionally, we further validated the anti-ZIKV effect of OTUD4 by downregulation through siRNA transfection. OTUD4 siRNA could efficiently suppress its mRNA and protein expression in A549 cells in the presence or absence of ZIKV infection (Figures 5D,F). As expected, silencing of OTUD4 significantly promoted ZIKV RNA replication and ZIKV NS1 protein level compared with negative control group (Figures 5E,F). Furthermore, to determine whether OTUD4 could counteract the pro-viral effect of miR-103a-3p on ZIKV replication, we co-transfected miR-103a-3p and OTUD4 plasmid into A549 cells in the presence of ZIKV infection. As expected, OTUD4 over-expression abrogated the stimulating effect of miR-103a-3p on ZIKV replication (Figures 5G,H). We further assessed the effect of OTUD4 on p38 MAPK pathway in ZIKV-infected A549 cells. We found OTUD4 over-expression inhibited the phosphorylation of both p38 MAPK and HSP27 (Figure 5C), while OTUD4 knock down upregulated the phosphorylation of p38 MAPK and HSP27 (Figure 5F). Moreover, OTUD4 attenuated the activation effect of miR-103a-3p on the p38 MAPK signaling pathway as shown by the decreased phosphorylation levels of both p-p38 MAPK and p-HSP27 in the presence of OTUD4 over-expression (Figure 5H). Besides, OTUD4 knock-down or over-expression did not affect cell viability (Figure 5I). Taken together, these findings indicate that OTUD4 not only inhibits ZIKV replication through suppression of p38 MAPK signaling pathway but also attenuates the stimulating effect of miR-103a-3p on ZIKV replication.

## Discussion

In the past few years, accumulating evidence has suggested that miRNAs regulate a variety of biological processes including host immune responses, apoptosis, and viral infections (Fani et al., 2018). Although miRNAs play important roles in the pathogenesis of *flavivirus* infections, only a few studies attempt to analyze how ZIKV infection alters host miRNA expression (Kozak et al., 2017; Dang et al., 2019; Seong et al., 2020; Tabari et al., 2020). Interestingly, we found different research groups showed significantly different results of the miRNAs expression profiles induced by ZIKV infection, which may be due to different ZIKV lineages, sample harvested time and cell lines used in different studies. ZIKV was reported to infect lung in animal models. One study showed that in a primate model, ZIKV was subcutaneously injected into pregnant rhesus macaques, and vRNA could be detected in the lungs of fetus (Nguyen et al., 2017). Another study demonstrated that primary cells from lung tissues of tree shrew permissive to ZIKV infection (Zhang et al., 2019b). In addition, human lung epithelial A549 cells have been shown to be permissive to infection by most flaviviruses including ZIKV (Frumence et al., 2016; Liao et al., 2020) and are a suitable model for studying host-virus interactions. In this study, we first performed a small RNA sequencing to identify dysregulated miRNAs profiles during the course of ZIKV (GZ01 strain) infection in A549 cells. As shown in Figure 1, although slightly differences within the three samples for each group were observed in cluster analysis, 35 miRNAs were statistically differentially regulated in ZIKV-infected A549 cells (*p* < 0.05). We further confirmed the expression levels of 10 miRNAs obtained by RNA-Seq using qRT-PCR, which were consistent with RNA-Seq results.

MiR-103a-3p is one of the upregulated miRNAs induced by ZIKV infection. A recent study also reported that miR-103a-3p was upregulated in EVs during ZIKV infection (Tabari et al., 2020). MiR-103a-3p has multiple functions involved in the regulation of inflammation, immune response, and development of a variety of cancers (Geng et al., 2014; Hu et al., 2018; Lu et al., 2019; Li et al., 2020, 2021). However, to date, the effect of miR-103a-3p on the regulation of pathogen infection has not been reported. Therefore, we focus on miR-103a-3p to explore the role of miR-103a-3p in ZIKV replication and its underlying mechanisms. We upregulated the expression level of miR-103a-3p by transfection to study its role in ZIKV replication. We found that over-expression of miR-103a-3p stimulated ZIKV replication both at NS5 mRNA and NS1 protein levels (Figure 2) without affecting the cell viability. These results indicated that miR-103a-3p may be one of the host genes ZIKV exploited to benefit its own replication.

P38 MAPK signaling pathway has been reported to be involved in the pathogenesis of many viruses (Sreekanth et al., 2016; Grimes and Grimes, 2020). It has been well documented that several viruses facilitate their replication by activating p38 MAPK signaling pathway (Chander et al., 2021). For example, SARS-CoV-2 may directly activate p38 MAPK signaling to induce overwhelming inflammation, as well as simultaneously utilizes p38 MAPK signaling pathway to facilitate its replication (Grimes and Grimes, 2020). Similarly, activated p38α has been shown to interact with HCV core protein to promote oligomerization of the HCV core protein, and subsequently facilitates HCV replication, while disruption of the p38α-core interaction by SB203580 inhibits HCV assembly. In addition, similar results were also observed in SFTSV and HSV-1 infections (Cheng et al., 2020). However, whether ZIKV infection activates p38 MAPK signaling is controversially. One earlier study reported that ZIKV infection activated p38 MAPK signaling pathway in Müller cells (Zhu et al., 2017). Another study also showed that ZIKV activated p38 MAPK in JAR, JEG-3, and HTR-8 cells (Muthuraj et al., 2021). However, one study failed to show any effect of ZIKV infection on p38 MAPK activity (Cheng et al., 2020). These earlier studies used different virus strains, cell lines, and sample harvesting time, which may contribute to different findings. In this current study, we confirmed that ZIKV infection induced p38 MAPK activation in both time-dependent and MOI-dependent manners (Supplementary Figure S1), while blockage of p38 MAPK activity by inhibitor SB203580 repressed ZIKV replication (Figure 3B). In addition, pretreatment of SB203580 alone did not affect miR-103a-3p in A549 cells, but slightly inhibited the ZIKV induced miR-103a-3p expression (Supplementary Figure S2), which indicated that ZIKV induced miR-103a-3p is associate with the ZIKV infection and independent of p38 MAPK pathway. Quite interestingly, we observed a slight increase in phosphorylation of p38 MAPK in the presence of SB203580 in A549 cells. Kumar et al. (1999) also observed similar phenomenon and speculated that it may be due to inaccessibility of phosphatase responsible for p38 MAPK dephosphorylation in the presence of SB203580. Alternatively, the p38 MAPK enzyme may be stabilized in a state that is more accessible to MKK6 due to SB203580 binding. Therefore, the binding of SB203580 in the ATP binding pocket of p38 MAPK interferes with its catalytic activity but does not inhibit p38 MAPK phosphorylation by upstream kinases. HSP27 is one of effector molecule downstream of the p38 MAPK signaling pathway and can be activated by virus infection (Mathew et al., 2009). It has been reported that adenovirus infection activates p38 MAPK, and HSP27, which is required for virus nuclear targeting (Suomalainen et al., 2001). HSP27 or p-HSP27 depletion can reduce HSV-1 production significantly (Mathew et al., 2009). These studies implied that HSP27 plays an important role in regulating virus infection.

Our data indicated that either ZIKV infection or miR-103a-3p alone could effectively activated p38 MAPK signaling pathway as shown by the increased level of p-p38 MAPK and p-HSP27 (Figure 3C). After we confirmed that miR-103a-3p could stimulate ZIKV replication and p38 MAPK signaling pathway was activated following ZIKV infection, we moved on to clarify whether the stimulating effect of miR-103a-3p on ZIKV replication is mediated by p38 MAPK signaling pathway. To this end, an inhibitor of p38 MAPK signaling pathway (SB203580) was used. We found the stimulating effect of miR-103a-3p on ZIKV replication was attenuated if the activation of p38 MAPK signaling pathway was inhibited by SB203580 (Figures 3D,E), which indicates that p38 MAPK signaling pathway is involved in the regulation of ZIKV replication by miR-103a-3p.

MicroRNAs normally function through downregulation of their target genes. In order to further understand the molecular mechanism by which miR-103a-3p stimulates ZIKV replication, we used four different prediction algorithms to predict the possible target genes of miR-103a-3p. As shown in Supplementary Figure S3, 177 overlapping genes predicted by all four algorithms were obtained. Among these genes, OTUD4 was previously reported to be associated with host antiviral response and p38 MAPK pathway (Zhao et al., 2018; Liuyu et al., 2019). As a k48-specific DUB, OTUD4 could stabilize MAVS by preventing its degradation to facilitate antiviral response (Liuyu et al., 2019). Furthermore, OTUD4 was identified as a phospho-activated k63 DUB, which targeted toll-like receptor (TLR)-associated factor MyD88 and negatively regulated TLR-mediated activation of NF-κB; Moreover, silencing of OTUD4 has been shown to enhance the phosphorylation of p38 MAPK in cells upon IL-1β stimulation (Zhao et al., 2018), which may indicate OTUD4 is a negative regulator of p38 MAPK signaling pathway. In our current study, we first identified a potential binding site for miR-103a-3p at the 3′UTR region of OTUD4 (Figure 4A). Using mutation analysis, we were able to confirm that OTUD4 is indeed one of the target genes of miR-103a-3p (Figure 4C). We further assessed the effect of OTUD4 on ZIKV replication and activation of p38 MAPK signaling pathway. As shown in Figure 5, over-expression of OTUD4 inhibited, while silencing of OTUD4 promoted ZIKV replication both at NS5 mRNA and NS1 protein levels. We also observed decreased phosphorylation of p38 MAPK and HSP27 in OTUD4 over-expressed cells, while increased in OTUD4 silenced cells in the presence or absence of ZIKV infection. Most importantly, OTUD4 attenuated the simulating effect of miR-103a-3p on ZIKV replication together with inhibition of p38 MAPK signaling pathway (Figures 5G,H).

**Figure 6:**
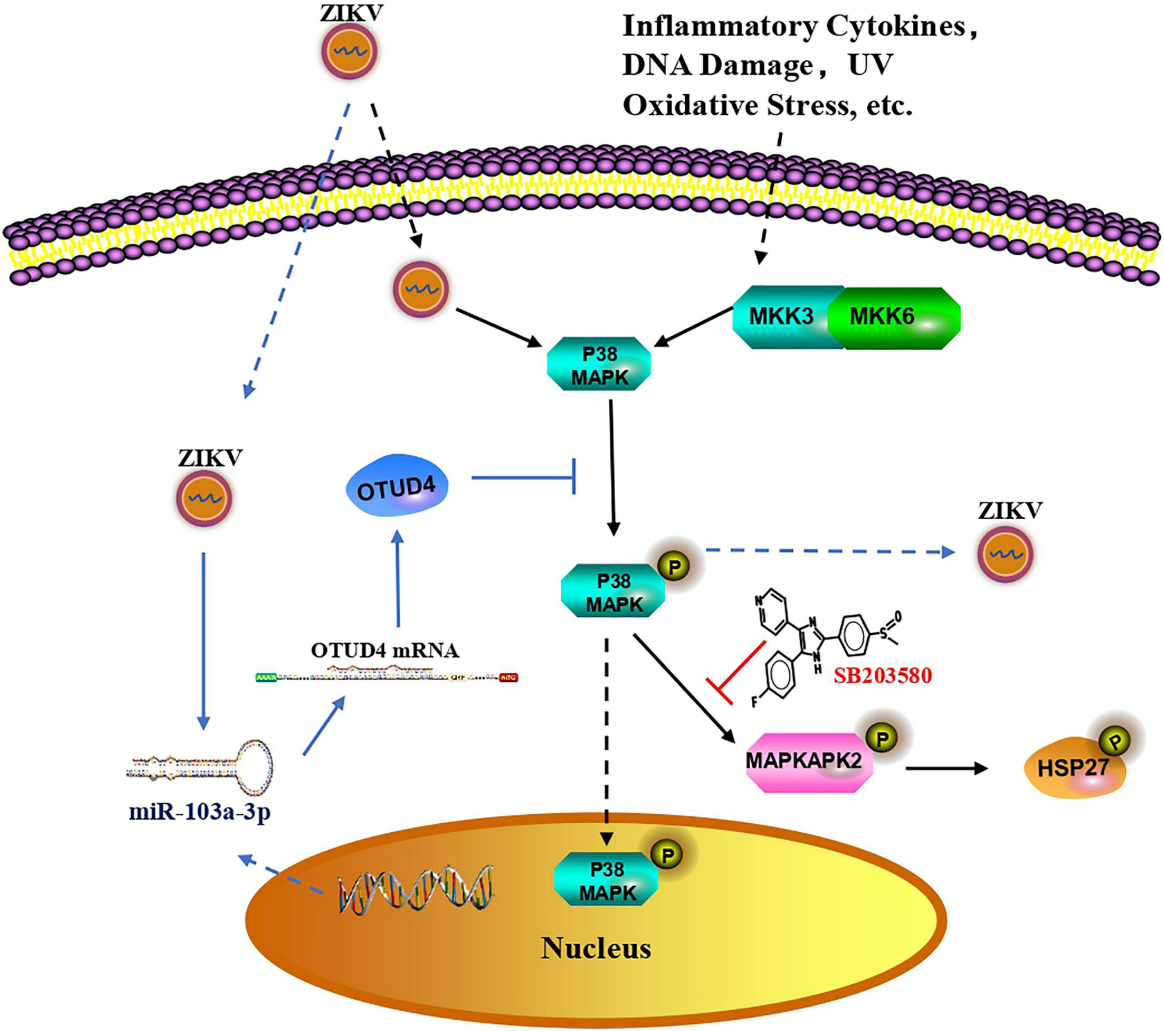
Proposed model for miR-103a-3p regulates ZIKV replication. In response to ZIKV infection, p38 MAPK is activated as shown by increased phosphorylated of HSP27 which facilitates ZIKV replication. Suppressed activity of p38 MAPK by SB203580 inhibits ZIKV replication. ZIKV infection also induces miR-103a-3p expression, which targets OTUD4 to regulate p38 MAPK signaling pathway to stimulate ZIKV replication. MiR-103a-3p is one of the host genes exploited by ZIKV to benefit its replication and targeting this gene or the p38 MAPK signaling pathway may be a potential novel strategy to treat ZIKV infection.

In summary, data from our current study indicated that ZIKV infection induced miR-103a-3p upregulation, which in turn was exploited by ZIKV to promote its own replication through targeting OTUD4 to activate p38 MAPK signaling pathway (Figure 6). Our research revealed a novel miRNA-mediated activation of the p38 MAPK signaling pathway to promote ZIKV replication, implying that miR-103a-3p is one of the host genes exploited by ZIKV to benefit its replication and targeting this gene or the p38 MAPK signaling pathway may be a potential novel strategy to treat ZIKV infection.

## Conclusion

We conclude that miR-103a-3p plays an important role in regulating ZIKV replication. MiR-103a-3p stimulates ZIKV replication by targeting OTUD4 to activate the p38 MAPK signaling pathway and targeting miR-103a-3p or p38 MAPK signaling may provide a viable approach for the treatment of ZIKV infection.

## Data Availability Statement

The datasets presented in this study can be found in online repositories. The names of the repository/repositories and accession number(s) can be found in the article/Supplementary Material.

## Author Contributions

HY, XD, and LC conceived and designed the project. HY, LK, XY, YH, and RM performed the experiments. HY, XD, and SL analyzed and interpreted the data. HY drafted the initial manuscript. SL, XD, and LC reviewed and critically revised the initial draft. All authors contributed to the article and approved the submitted version.

## Funding

This work is supported by Natural Science Foundation of China (NSFC 82102383) and the central government directed special funds for local science and technology development project (No 2021ZYD0085) to XD; by National Key Research and Development Program of China (2018YFE0107500), and the Science and Technology Partnership Program, Ministry of Science and Technology of China (KY201904011) to LC.

## Conflict of Interest

The authors declare that the research was conducted in the absence of any commercial or financial relationships that could be construed as a potential conflict of interest.

## Publisher’s Note

All claims expressed in this article are solely those of the authors and do not necessarily represent those of their affiliated organizations, or those of the publisher, the editors and the reviewers. Any product that may be evaluated in this article, or claim that may be made by its manufacturer, is not guaranteed or endorsed by the publisher.

## Footnotes

^1^ https://www.ncbi.nlm.nih.gov/geo/query/acc.cgi?acc=GSE146423

^2^ http://www.pictar.org/

^3^ http://www.targetscan.org/

^4^ http://microrna.sanger.ac.uk/

^5^ https://mirmap.ezlab.org/

^6^ https://bioinfogp.cnb.csic.es/tools/venny/index.html

## Supplementary Material

Supplementary Figure S1 Zika virus (ZIKV) infection activates p38 mitogen-activated protein kinase (MAPK) signaling pathway in A549 cells. ZIKV infected A549 cells at indicated multiplicity of infection (MOI) and harvested cells at indicated time post infection. **(A)** ZIKV infection increases p-p38 MAPK protein level in a time-dependent manner. **(B)** ZIKV infection enhances p-p38 MAPK protein level in a MOI-dependent manner at 48 h post infection.

Supplementary Figure S2 Zika virus -induced miR-103a-3p expression is slightly inhibited by SB203580 pretreatment. A549 cells were pretreated with SB203580 for 1 h and infected with ZIKV at a MOI of 0.5. Cells were harvested at 48 h post infection. SB20350 inhibited ZIKV-induced miR-103a-3p expression. Data are presented as mean ± SD. Error bars indicate SD (*n* ≥ 3). ^**^*p* < 0.01 and ^***^*p* < 0.001.

Supplementary Figure S3 Bioinformatics analysis of miR-103a-3p target gene. Four different prediction algorithms were used to predict the putative miR-103a-3p targets. Around 177 targets genes are overlapped in four prediction datasets.
