# Supplementary figures and images for: MiR-103a-3p Promotes Zika Virus Replication by Targeting OTU Deubiquitinase 4 to Activate p38 Mitogen-Activated Protein Kinase Signaling Pathway
Source: Front Microbiol. 2022 Mar 4;13:862580. doi: 10.3389/fmicb.2022.862580 (PMC8934420; doi:10.3389/fmicb.2022.862580)

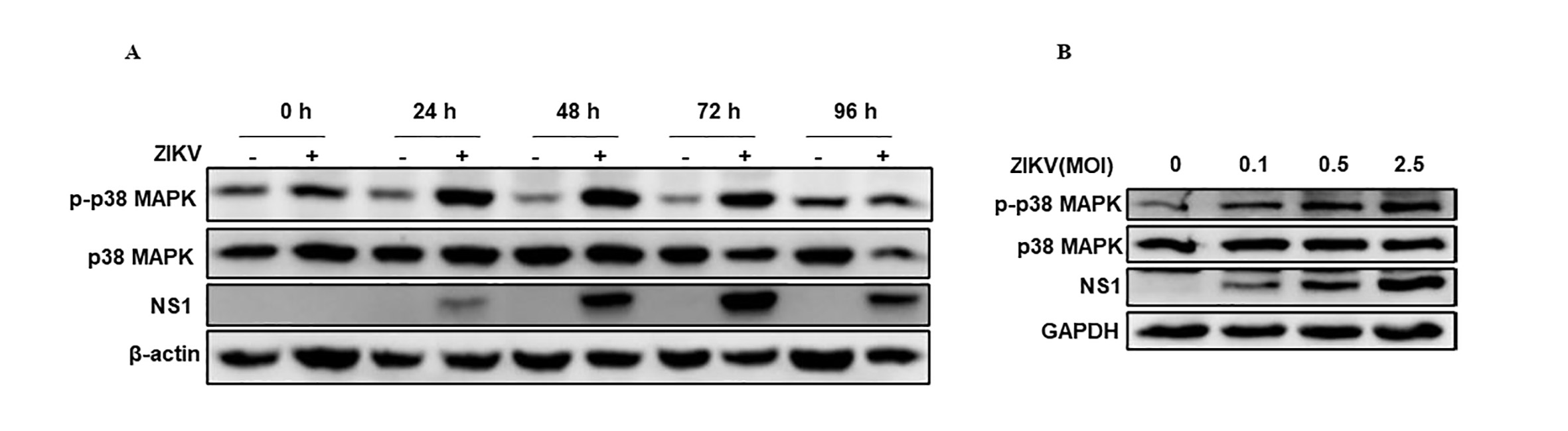

Supplement: Supplementary Figure S1 — Zika virus (ZIKV) infection activates p38 mitogen-activated protein kinase (MAPK) signaling pathway in A549 cells. ZIKV infected A549 cells at indicated multiplicity of infection (MOI) and harvested cells at indicated time post infection. (A) ZIKV infection increases p-p38 MAPK protein level in a time-dependent manner. (B) ZIKV infection enhances p-p38 MAPK protein level in a MOI-dependent manner at 48 h post infection. [file Image_1.JPEG]

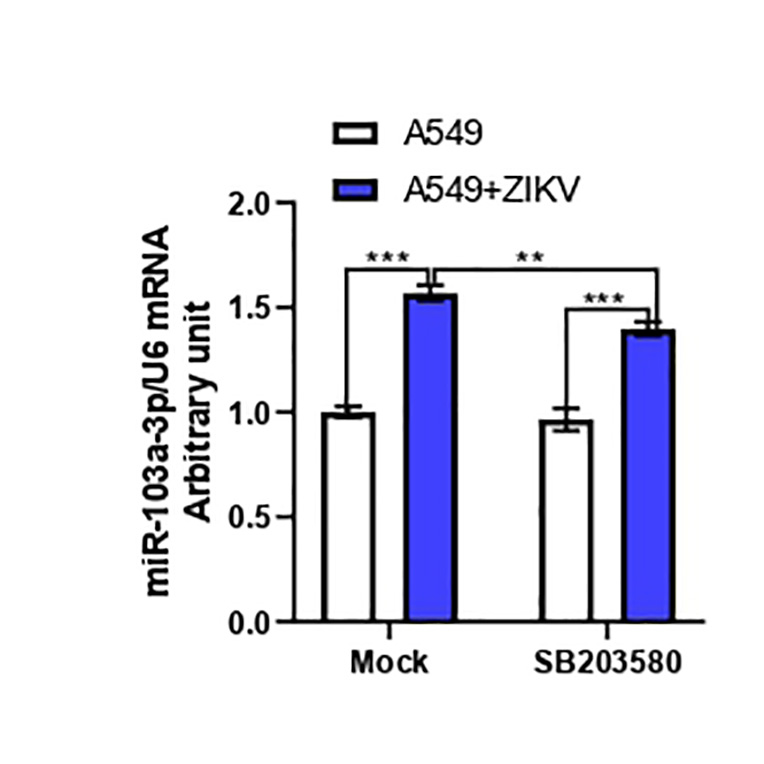

Supplement: Supplementary Figure S2 — Zika virus -induced miR-103a-3p expression is slightly inhibited by SB203580 pretreatment. A549 cells were pretreated with SB203580 for 1 h and infected with ZIKV at a MOI of 0.5. Cells were harvested at 48 h post infection. SB20350 inhibited ZIKV-induced miR-103a-3p expression. Data are presented as mean ± SD. Error bars indicate SD (n ≥ 3). **p < 0.01 and ***p < 0.001. [file Image_2.JPEG]

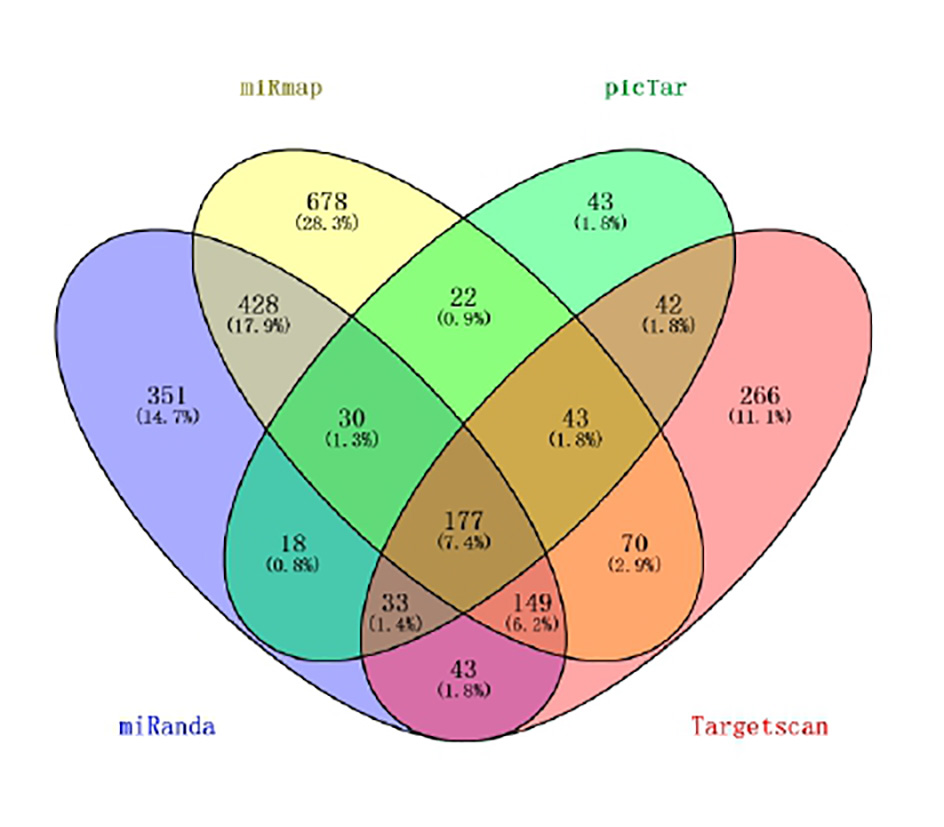

Supplement: Supplementary Figure S3 — Bioinformatics analysis of miR-103a-3p target gene. Four different prediction algorithms were used to predict the putative miR-103a-3p targets. Around 177 targets genes are overlapped in four prediction datasets. [file Image_3.JPEG]
